# The efficacy of pericapsular nerve group block for reducing pain and opioid consumption after total hip arthroplasty: a systematic review and meta-analysis

**DOI:** 10.1186/s13018-024-04707-x

**Published:** 2024-04-08

**Authors:** Chunjie She, Hefeng Liu

**Affiliations:** Department of Orthopaedics, Chaohu Hospital Affiliated to Anhui Medical University, Chaohu, Anhui China

**Keywords:** Opioid, Pericapsular nerve group block, Pain, Total hip arthroplasty

## Abstract

**Background:**

Pericapsular nerve group block (PENG) is an emerging regional anesthesia technique for hip surgery. However, its efficacy in total hip arthroplasty (THA) isn’t well defined. We perform this meta-analysis aiming to assess the effect of Pericapsular nerve group block on pain control and morphine consumption in patients with total hip arthroplasty.

**Methods:**

We searched four electronic databases (Pubmed, Embase, Cochrane Library, and Web of Science dated from 2018 to October 2023) for published eligible randomized controlled trials (RCTs) comparing PENG with placebo (no block/sham block) after THA. The outcome measurements consisted of pain score, opioid consumption, Time to first opioid, and postoperative complications. All data analyses were performed using STATA 12.0.

**Results:**

Five RCTs comprising 808 participants were included. Our meta-analysis showed that there were significant differences between two groups in terms of pain score in PACU (WMD =  − 0.598, 95% CI [− 0.886, − 0.310], *P* < 0.001), pain score at 6 h (WMD =  − 0.614, 95% CI [− 0.835, − 0.392], *P* < 0.001) and time to first opioid (WMD = 5.214, 95% CI [4.545, 5.883], *P* < 0.001). However, no significant differences were revealed from the pain score at 24 h after THA (WMD =  − 0.924, 95% CI [− 1.929, 0.081], *P* = 0.072). Meanwhile, the meta-analysis indicated that PENG significantly reduced 24-h opioid consumption (WMD =  − 6.168, 95% CI [− 6.667, − 5.668], *P* < 0.001) and 48-h opioid consumption (WMD =  − 7.171, 95% CI [− 8.994, − 5.348], *P* < 0.001).

**Conclusion:**

Pericapsular nerve group block was effective for pain control up to postoperative 6 h and extending the time to the first opioid after THA. Moreover, it reduced postoperative opioid consumption when compared with a placebo group. Due to the high heterogeneity of the pain score after 24 h and the low-quality evidence, more high-quality RCTs are required to draw a definitive conclusion about pain control.

**Supplementary Information:**

The online version contains supplementary material available at 10.1186/s13018-024-04707-x.

## Introduction

Total hip replacement is a cost-effective surgical procedure used to reduce pain and restore the function of arthritic hip joints. More than 1 million joint replacements are performed worldwide each year, and this number is expected to double in the next 20 years [1]. However, it is usually accompanied by moderate to severe pain after surgery [2]. Severe postoperative pain can affect patients' normal functional rehabilitation training, prolong the time to hospital discharge, and increase the risk of postoperative complications such as lower limb thrombosis and pneumonia [3, 4]. At present, a variety of analgesic methods can be used as postoperative analgesia methods in THA, including patient-controlled opioid analgesia, local infiltration analgesia around the joint, epidural analgesia, and peripheral nerve block [5]. Regional nerve blocks may also be used as a component of a multimodal analgesic protocol to manage postoperative pain after primary total hip arthroplasty [6]. Pericapsular nerve group block (PENG) is a new regional block proposed by Giron-Arango et al. [7]. It was initially used for the early analgesia of hip fractures and has been proven to be an effective analgesia for acute traumatic pain [8]. PENG relieves pain by blocking the obturator nerve (ON), accessory obturator nerve (AON), and femoral nerve of the anterior capsule of the hip, which is theoretically less likely to cause quadriceps block than some peripheral nerve blocks such as the femoral nerve block (FNB) and fascia iliaca compartment block (FICB) [9–11]. By comparing pericapsular nerve block anesthesia with other different regional nerve block anesthesia in hip fractures, Liang Yu and Wang Yi's meta-analysis reported that PENG block provides effective analgesia, similar to FICB and other regional nerve blocks in hip surgery [3, 12]. However, these meta-analyses, like most of the published literature about pericapsular nerve group block, included different types of hip surgery and different treatment of controls, so the results should be viewed with caution.

Therefore, we conducted a meta-analysis of randomized controlled trials (RCTS) that used placebo as a control group and included THA only. Compared with placebo, we hypothesized that PENG was associated with pain relief and morphine sparing.

## Methods

This systematic review and meta-analysis has been reported in line with PRISMA (Preferred Reporting Items for Systematic Reviews and Meta-Analyses) Guidelines and was prospectively registered on the International Prospective Register of Systematic Reviews (PROSPERO –CRD42023486844).

### Search strategy

We searched four electronic databases including Pubmed, EMBASE, Cochrane Library, and Web of Science from 2018 to October 2023, using medical subject headings (MeSH) and free-text terms without language restrictions. The search strategy was as follows: “Nerve Block” and “Arthroplasty, Replacement, Hip”. Since the PENG is a newly regional anesthesia technique, first reported in 2018, we chose a wider range of (MeSH) and free-text terms to avoid omission. The database search was completed by two independent researchers to reach a consensus. If a consensus was not reached, a third reviewer would make a judgment (Additional file 1).

### Inclusion criteria and exclusion criteria

The inclusion criteria were as follows: (a) patients undergoing THA; (b) received PENG as the intervention treatment (PENG group), and no block or sham block as the placebo treatment (control group); (c) at least one of the following outcome measures was reported: postoperative pain score, opioid consumption;(d) randomized controlled trials. Exclusion criteria were as follows: (a) reviews, replies, letters, case reports, and non-randomized studies, including retrospective design and incomplete clinical trials; (b) unavailable data for extraction.

### Data extraction

Two authors independently screened the final enrolled RCTs and collected the following data: first author, publication year, country, sample size, patient characteristics, type of surgery, type of anesthesia, treatment of PENG block group, treatment of control group, and outcome measurements. We extracted the mean and standard deviation (SD) of continuous or ordinal variables (opioid consumption or pain score) and the number (incidence) of dichotomous variables (postoperative complications). We contacted the corresponding author via email to collect outcome measurements as completely as possible. For part data that did not receive any response from the corresponding author, we estimated the mean as equivalent to the median and used the range and median values to estimate standard deviation based on the methods described by Wang et al. [13]. All values of opioid consumption were converted to intravenous morphine milligram equivalents according to a standard conversion table [14]. We calculated all data with the guidelines of the Cochrane Handbook for Systematic Reviews of Interventions 5.1.0. The included studies assessed the pain score using either the visual analogue scale (VAS) or the numeric rating scale (NRS). The VAS and NRS for pain were regarded as equivalent [15]. Disagreements were resolved through discussions.

### Quality assessment of included studies

The Cochrane Collaboration “Risk of bias” tool was used to evaluate the methodological quality. Seven items were assessed as follows: (I) random sequence generation, (II) allocation concealment, (III) blinding of participants and personnel, (IV) blinding of outcome assessment, (V) incomplete outcome data, (VI) selective reporting, and (VII) other bias. Each item was defined as a low risk, high risk, or unclear risk. Recommendations Assessment, Development, and Evaluation (GRADE) system was used to grade the evidence level. Five factors were considered as follows: (i) risk of bias, (ii) Inconsistency, (iii) indirectness, (iv) imprecision, (v) Publication bias. The quality of evidence was classified as high quality, medium quality, low quality, or very low quality.

### Statistical analysis

All data analyses were conducted by STATA 12.0. Weighted mean differences (WMD) with 95% confidence intervals (CIs) were calculated for continuous variables (e.g. opioid consumption or pain score) and Odds ratio (OR) with a 95% CI was calculated for dichotomous outcome. (e.g. postoperative complications). And *p* value < 0.05 with 95% CI was considered statistically significant. Heterogeneity was examined using I^2^ statistic. Studies with an I^2^ < 50% were considered to have low heterogeneity and a fixed-effect model was used. I^2^ > 50% were considered high heterogeneity, and we used a random-effects model. Sensitivity analyses were performed via the leave-one-out approach to evaluate whether the results were changed significantly when excluding a single trial. Potential publication bias was identified by the funnel plot, Egger's regression, and Begg’s test.

## Results

### Literature search

**Fig. 1:**
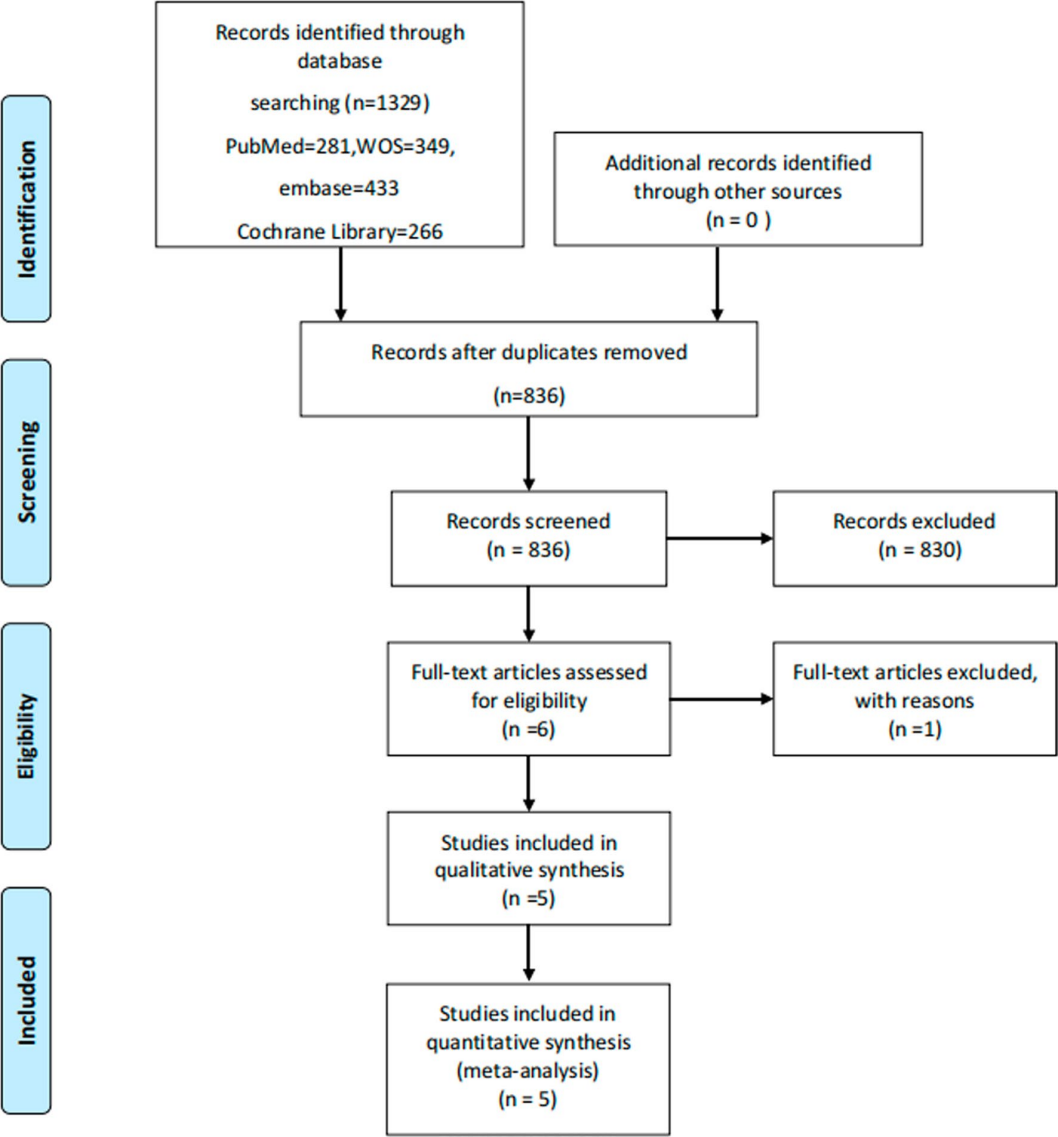
Flow chart of the study selection process

The PRISMA statement flowchart shows the process of literature screening (Fig. 1). A total of 1329 relevant articles were retrieved from database searches, of which 493 were excluded due to duplication. Based on the titles and abstracts, 712 were excluded for irrelevant articles. And 118 works of literature such as reviews, replies, letters, and case reports were excluded. In addition, by manually reviewing full-text, one of the remaining 6 pieces of literature was excluded for data that was not available. Finally, five randomized controlled trials were met our inclusion criteria and performed in this meta-analysis [16–20].

### Study characteristics participants

All of these RCTs were published between 2021 and 2023, involving 808 participants with surgery of total hip arthroplasty. Experiential groups received a pericapsular nerve group block for postoperative pain management and control groups received a placebo. The mean age ranged from 59-year-old to 66-year-old in PENG groups and 56-year-old to 67-year-old in control groups. Two groups received general anesthesia and the remaining three received spinal anesthesia. The trial characteristics are presented in Table 1.

**Table 1:** The details of trial characteristics

Author, year	Country	Design	Study	No. of patients		Gender (male)		Age		Anesthesia method	Treatment		Outcome
				PENG	Control	PENG	Control	PENG	Control		PENG	Control	
Kukreja, 2023	USA	RCT	THA	56	56	26	22	59	62	Spinal anesthesia	bupivacaine 0.5% (25 ml)	no block	1, 2, 3, 4, 6, 7
Hu, 2023	China	RCT	THA	45	45	21	23	59	56	General anesthesia	Ropivacaine (20 mL, 0.5%) containing 1:200,000 epinephrine	20 mL 0.9% saline	1, 2, 3, 4, 5, 6, 7, 8
Domagalska, 2023	Poland	RCT	THA	239	237	140	122	66	66	Spinal anesthesia	20 mL of 0.5% ropivacaine	20 mL of 0.9% NaCl	3, 4, 5, 6, 7
Zheng,2022	China	RCT	THA	34	36	12	15	63	64	General anesthesia	20 mL 0.5% ropivacaine	20 mL 0.9% saline	1, 2, 3, 4, 7, 8
Pascarella,2021	Italy	RCT	THA	30	30	16	17	66	67	Spinal anesthesia	20 ml of ropivacaine 0.375%	no block	5, 7, 8

1, Pain score in PACU. 2, Pain score at 6 h. 3, Pain score at 24 h. 4, Pain score at 48 h. 5, Time to first opioid. 6, Opioid consumption at 24 h. 7, Opioid consumption at 24 h. 8, Incidence of complications

### Risk of bias

**Fig. 2:**
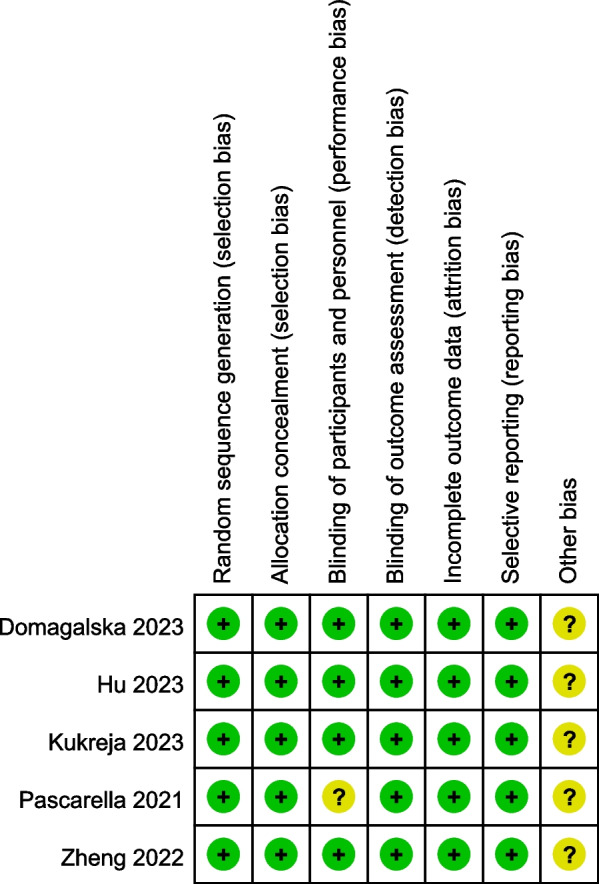
Risk of bias summary

**Fig. 3:**
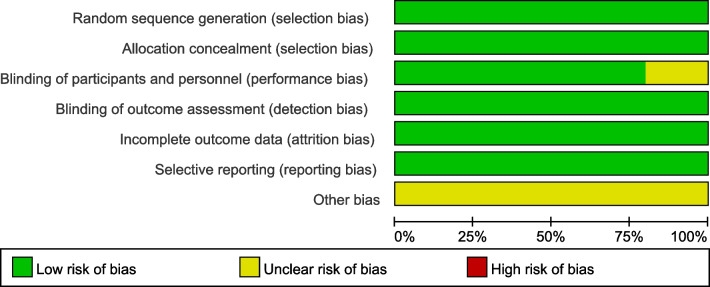
Risk of bias graph

The risk of bias summary and risk of bias graph are expressed in Figs. 2 and 3, respectively. As is shown in the two figures, most assessment of bias was categorized as low risk. One study involving blinding for participants and personnel was classified as unclear risk, and other assessments of bias were also unclear.

### Outcome of meta-analysis

#### Pain score in PACU

**Fig. 4:**
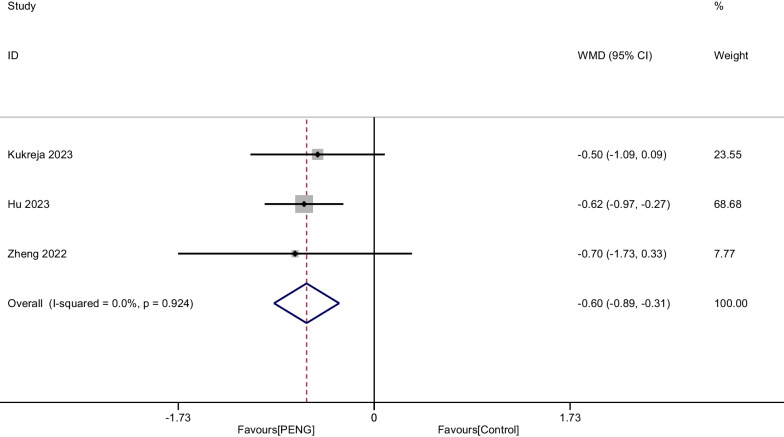
Forest plot diagram of pain score in PACU

Three RCTs including 272 patients reported the pain score in PACU. Since no significant heterogeneity was observed, we used a fixed-effect model (*P* = 0.924, I^2^ = 0%). The Pooled analysis demonstrated that PENG could significantly reduce the pain score in PACU after THA (WMD =  − 0.598, 95% CI [− 0.886, − 0.310] *P* < 0.001, Fig. 4).

#### Pain score at 6 h

**Fig. 5:**
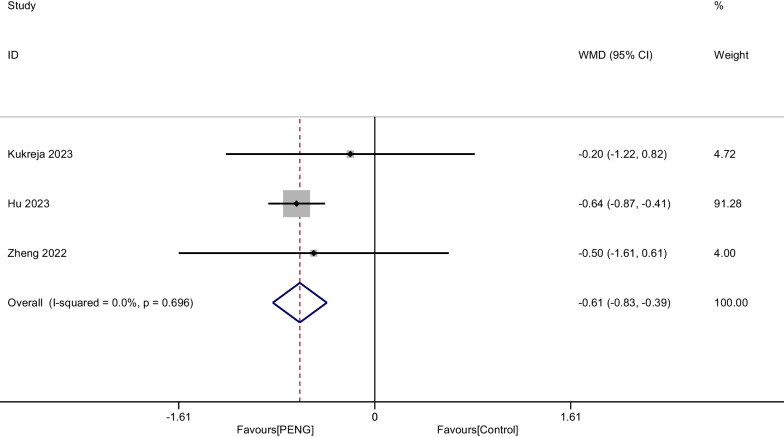
Forest plot diagram of pain score at 6 h

A total of three RCTs involving 272 patients showed pain at 6 h after THA. There was no significant heterogeneity and a fixed-effect modal was adopted. (*P* = 0.696, I^2^ = 0%). The aggregated results of these studies suggest that there was a significant difference between groups concerning pain at 6 h (WMD =  − 0.614, 95% CI [− 0.835, − 0.392], *P* < 0.001, Fig. 5).

#### Pain score at 24 h

**Fig. 6:**
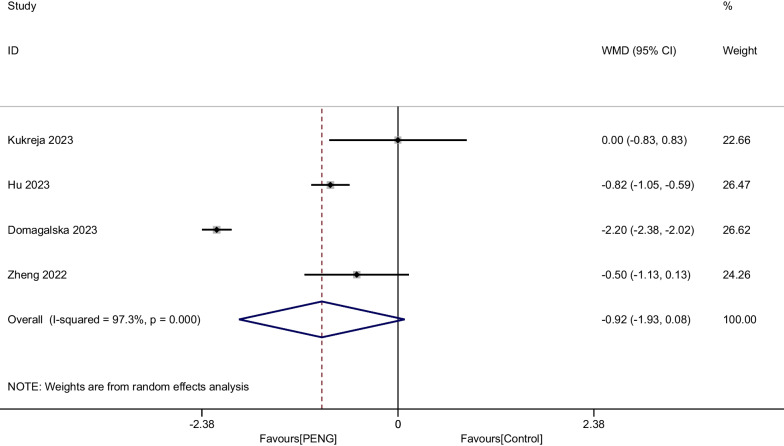
Forest plot diagram of pain score at 24 h

Four studies including 744 patients provided the outcome of pain at 24 h after THA. There was significant heterogeneity (*P* < 0.001, I^2^ = 97.3%), and a random-effects modal was adopted. The present meta-analysis demonstrated that there was no significant difference between groups regarding the pain at 24 h (WMD =  − 0.924, 95% CI [− 1.929, 0.081], *P* = 0.072, Fig. 6).

#### Pain score at 48 h

**Fig. 7:**
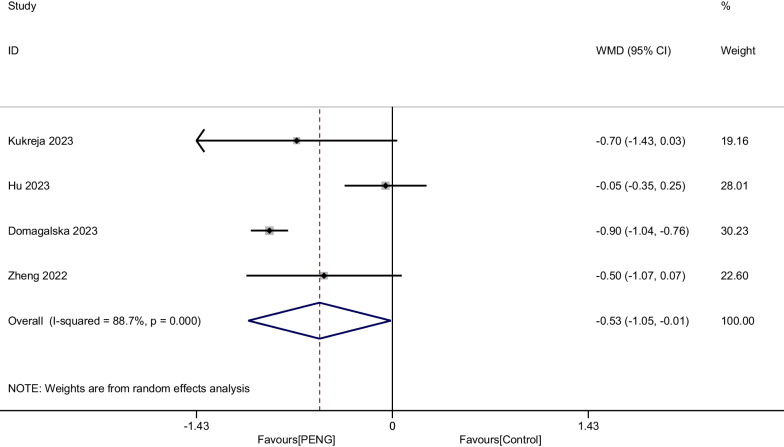
Forest plot diagram of pain score at 48 h

Four RCTs involving 744 patients compared the difference in the efficacy of PENG and placebo group on pain at 24 h. Significant heterogeneity was identified in the pooled results, therefore a random-effects model was used (I^2^ = 88.7%, *P* < 0.001). Our study indicated that there was a significant difference between the two groups (WMD =  − 0.533, 95% CI [− 1.055, − 0.012], *P* = 0.045, Fig. 7).

#### Time to first opioid

**Fig. 8:**
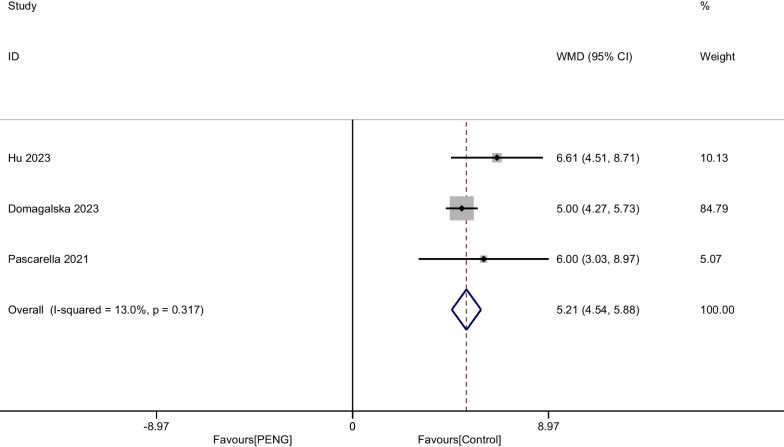
Forest plot diagram of time to the first opioid

Three studies involving 626 patients reported the outcome of time to the first opioid after THA. There was no heterogeneity among the included studies, therefore a fixed-effect model was used (I^2^ = 13.0%, *P* = 0.317). Our meta-analysis demonstrated that there was a significant difference between groups (WMD = 5.214, 95% CI [4.545, 5.883], *P* < 0.001, Fig. 8).

#### Opioid consumption at 24 h

**Fig. 9:**
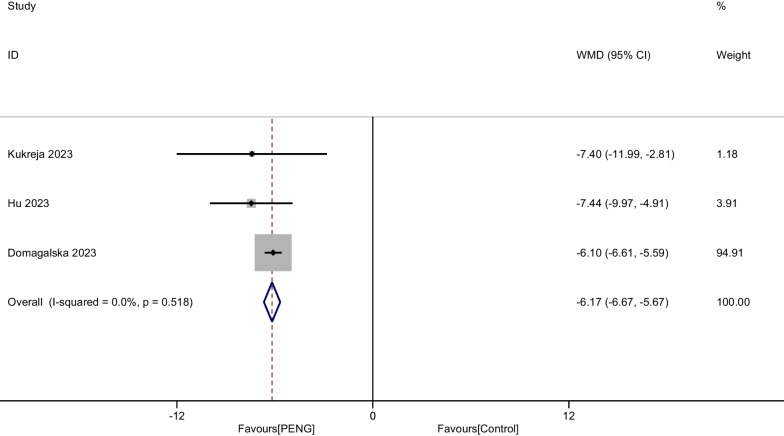
Forest plot diagram of opioid consumption at 24 h

Three RCTs including 678 patients provided the outcome of Opioid consumption at 24 h after THA. Due to no significant heterogeneity being found, a fixed-effect model was used (I^2^ = 0.0%, *P* = 0.518). Compared with the control group, PENG was associated with a significant reduction of Opioid consumption at 24 h (WMD =  − 6.168, 95% CI [− 6.667, − 5.668], *P* < 0.001, Fig. 9).

#### Opioid consumption at 48 h

**Fig. 10:**
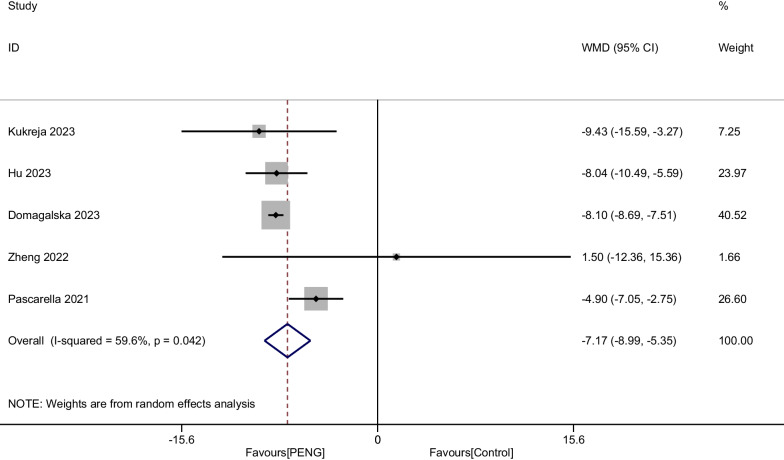
Forest plot diagram of opioid consumption at 48 h

All five RCTs involving 808 patients showed Opioid consumption at 48 h after THA. We used a random-effects model, with significant heterogeneity identified (*P* = 0.042, I^2^ = 59.6%). Our meta-analysis demonstrated that PENG could significantly reduce Opioid consumption at 48 h (WMD =  − 7.171, 95% CI [− 8.994, − 5.348], *P* < 0.001, Fig. 10).

#### Incidence of complications

**Fig. 11:**
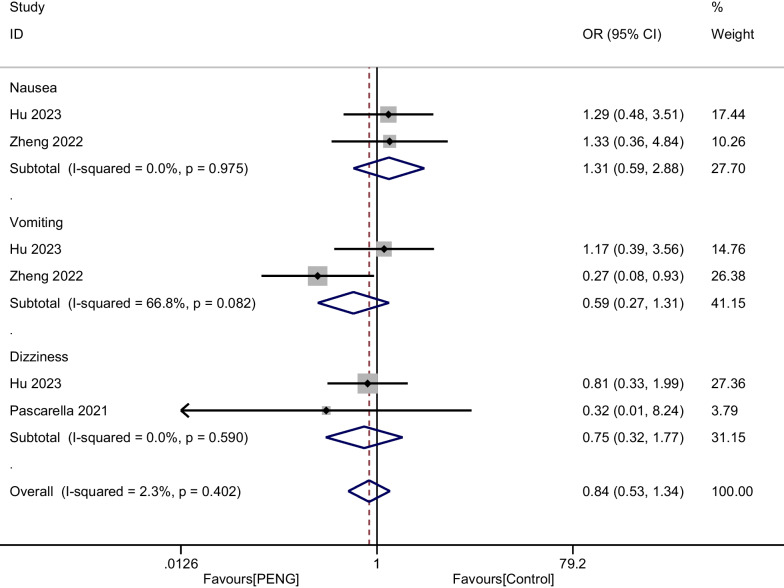
Forest plot diagram of incidence of complications

A total of three RCTs including 220 patients reported the postoperative complications after THA. There was no significant heterogeneity (*P* = 0.402, I^2^ = 2.3%), and a fixed-effect modal was used. The aggregated results showed that PENG neither reduced the incidence of nausea and vomiting nor was associated with an increase in the risk of dizziness (OR = 0.840, 95% CI [0.528, 1.366], *P* = 0.402, Fig. 11).

### Quality of evidence

The quality of evidence for the main outcomes varied from low to high, which indicated that further research was likely to significantly alter confidence in the effect estimate and may change the estimate. The details of the assessment are shown in Table 2.

**Table 2:** The GRADE evidence quality

Outcome	Number of studies/patients	Effect	Limitations	Inconsistency	Indirectness	Imprecision	Quality
Pain score in PACU	3/272	WMD = −0.598, 95% CI [− 0.886, −0.310]	No serious limitations	No serious inconsistency	No serious indirectness	No serious limitations	High
Pain score at 6 h	3/272	WMD = −0.614, 95% CI [− 0.835, −0.392]	No serious limitations	No serious inconsistency	No serious indirectness	No serious limitations	High
Pain score at 24 h	4/744	WMD = −0.924, 95% CI [− 1.929, 0.081]	No serious limitations	Serious inconsistency	No serious indirectness	Serious limitations	Low
Pain score at 48 h	4/744	WMD = −0.533, 95% CI [− 1.055, −0.012]	No serious limitations	Serious inconsistency	No serious indirectness	Serious limitations	Low
Time to first opioid	3/626	WMD = 5.214, 95% CI [4.545, 5.883]	No serious limitations	No serious inconsistency	No serious indirectness	No serious limitations	High
Opioid consumption at 24 h	3/678	WMD = − 6.168, 95% CI [− 6.667, − 5.668]	No serious limitations	No serious inconsistency	No serious indirectness	No serious limitations	High
Opioid consumption at 48 h	5/808	WMD = − 7.171, 95% CI [− 8.994, − 5.348]	No serious limitations	Serious inconsistency	No serious indirectness	No serious limitations	Moderate
Incidence of complications	3/220	OR = 0.840, 95% CI [0.528, 1.336]	No serious limitations	No serious inconsistency	No serious indirectness	No serious limitations	High

### Subgroup analyses, sensitivity analysis, and publication bias

**Fig. 12:**
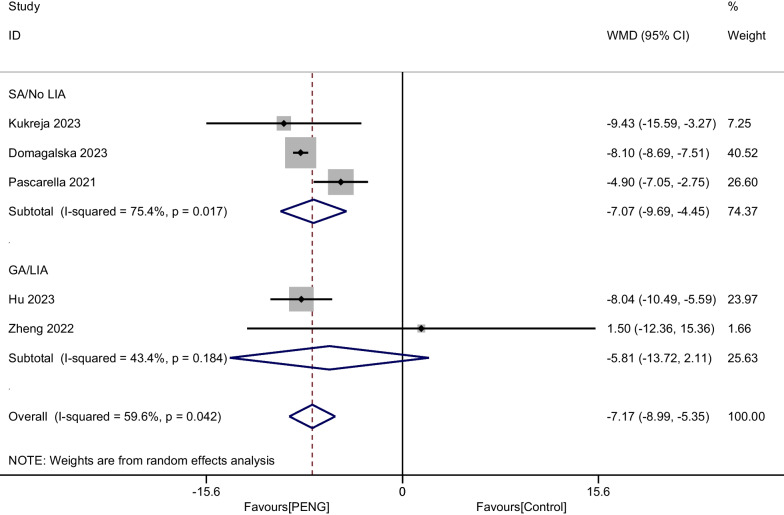
Subgroup analysis of whether combined with Local infiltration anesthesia (LIA) and the method of anesthesia (SA: Spinal anesthesia or GA: General anesthesia)

**Fig. 13:**
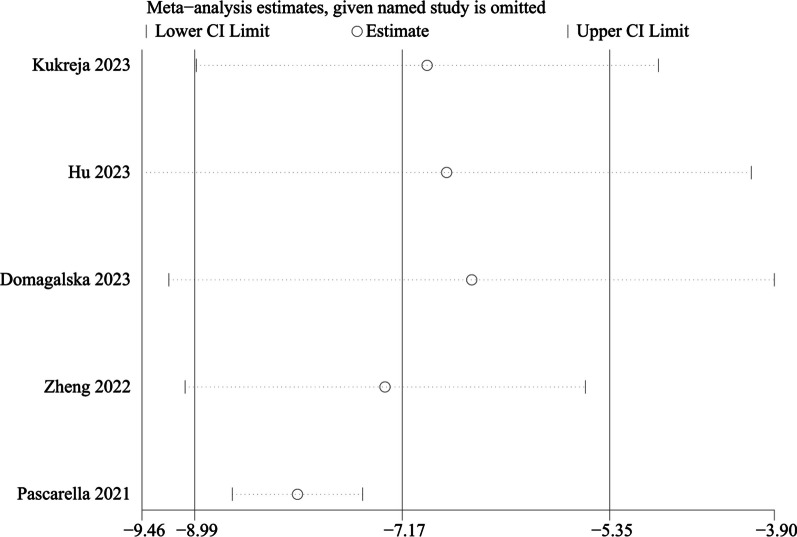
Sensitivity analysis of opioid consumption at 48 h

**Fig. 14:**
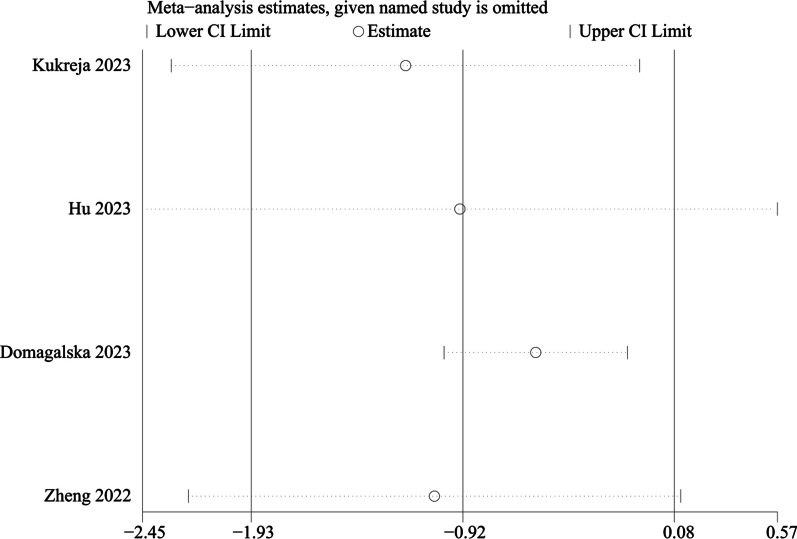
Sensitivity analysis of pain score at 24 h

**Fig. 15:**
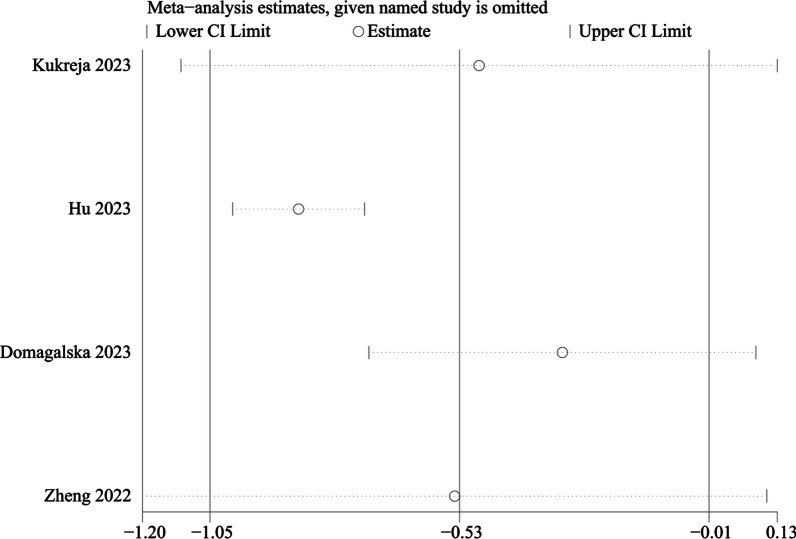
Sensitivity analysis of pain score at 48 h

**Fig. 16:**
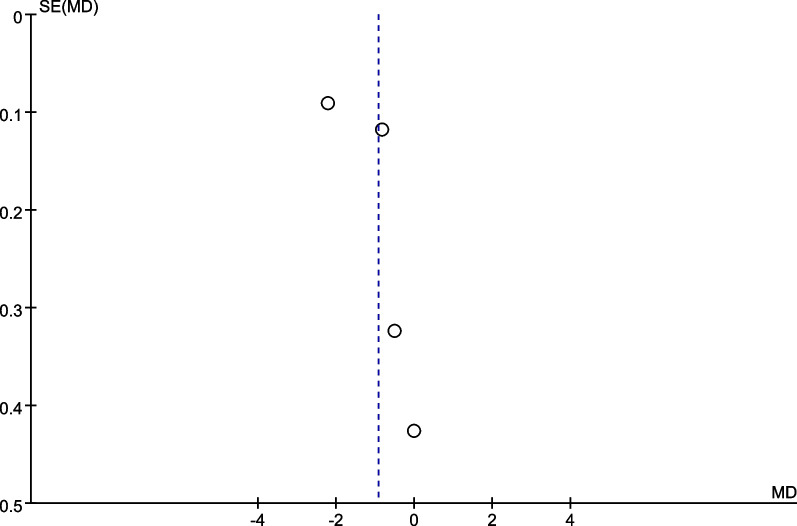
Funnel plot for pain score at 24 h

**Fig. 17:**
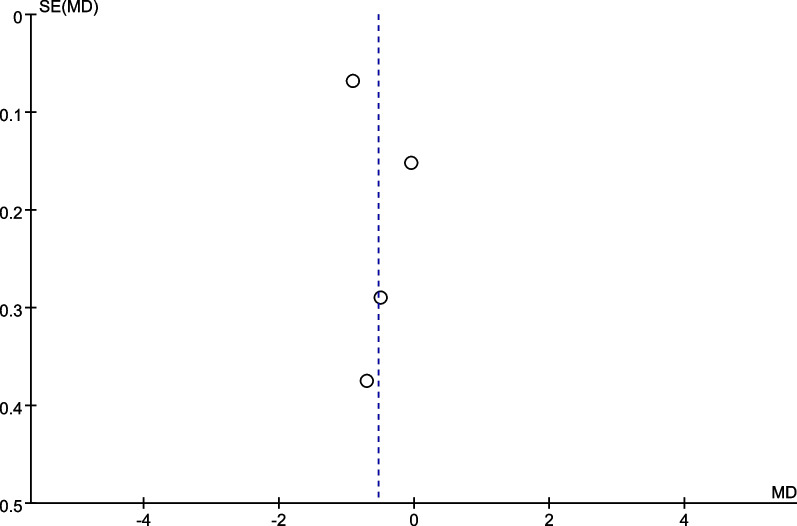
Funnel plot for pain score at 48 h

**Fig. 18:**
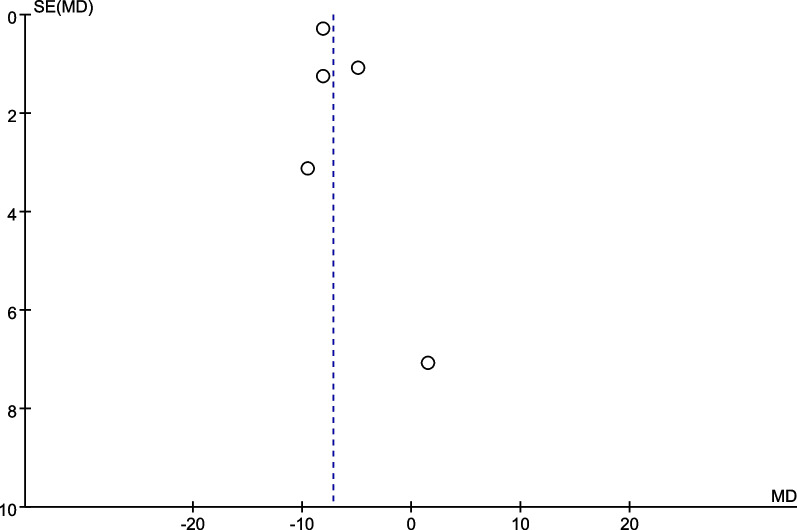
Funnel plot for opioid consumption at 48 h

We performed a subgroup analysis of whether combined with local infiltration anesthesia and the method of anesthesia, and no significant differences were found in the subgroup analysis between combined with infiltration anesthesia or not and different anesthesia methods (*P* = 0.77) (Fig. 12). Owing to the significant heterogeneity in pain score at 24 h (I^2^ = 97.3%), pain score at 48 h (I^2^ = 88.7%), and opioid consumption at 48 h (I^2^ = 59.6%), the sensitivity analysis was performed in our meta-analysis to confirm the stability of these results. Sensitivity analysis was performed by excluding one trial at a time and recalculating the pooled SMD for the remaining trials. Sensitivity analysis suggests that for opioid consumption at 48 h, none of the studies affected the most of the results, indicating a stable result (Fig. 13). However, in terms of pain score at 24 h (Fig. 14) and pain score at 48 h (Fig. 15), results were inconsistent. the funnel plots constructed with pain score at 24 h (Fig. 16), 48 h (Fig. 17), and opioid consumption at 48 (Fig. 18) were symmetrical, indicating a low risk of publication bias. At the same time, Egger's regression showed no significant publication bias (*P* = 0.358, *P* = 0.363, *P* = 0.447). Begg’s test also showed the same result (*P* = 0.806, *P* = 0.734, *P* = 1.0). However, publication bias was a limitation that existed in all meta-analyses.

## Discussion

### Principal findings

Our meta-analysis showed that PENG reduced postoperative pain scores in PACU and at 6 h after THA, but it may not be effective for pain relief after 24 h. The evidence for pain score after 24 h was low quality and with high heterogeneity, suggesting that further research was likely to significantly alter confidence in the effect estimate and may change the estimate. Moreover, PENG was associated with extending the time to the first opioid and reducing total morphine consumption. PENG was not associated with an increase in the risk of complications after THA when compared with placebo.

### Relationship to other systematic reviews

Previous systematic reviews evaluating the efficacy of PENG for postoperative analgesia have been published. According to a meta-analysis reported by Liang Yu, the PENG block compared with other regional nerve blocks provided an effective analgesic effect in hip surgery. Another meta-analysis performed by Wang et al. using FICB as a control group revealed that PENG block could reduce opioid use after hip surgery and is effective in postoperative analgesia. Although the research purpose of our meta-analysis was consistent with previous meta-analyses, differences between our study and previous studies should be considered. These two meta-analysis studies included all types of hip surgeries, which means that the heterogeneity of these two meta-analyses was higher than the study that only focused on total hip replacement. Second, In contrast to these previous meta-analyses, which used FICB or other nerve block anesthesia as a control group, our meta-analysis only included a placebo as a control. It is more direct to reflect the effect of pain relief and opioid consumption sparing. Our meta-analysis provides a new perspective for evaluating the analgesic effects of PENG compared to the previous meta-analyses.

### Implications for clinical practice

Our meta-analysis showed that PENG was effective for pain relief during the early postoperative period after total hip arthroplasty. We noticed that the pooled result of postoperative pain reduction and morphine reduction in published relevant meta-analysis is consistent with ours. However, the following two points require our attention before drawing conclusions. Based on the meta-analysis of Gao et al. and Cai et al. [21, 22]. On the analgesic effect of FICB after total hip replacement, there was a significant difference between placebo and FICB regarding the pain score at 12 h. According to the results of previous meta-analyses about PENG, PENG and FICB were similar in postoperative pain control [3, 12]. On the basis of the above two conclusions, we may conclude that there was a significant difference between placebo and PENG regarding the pain score at 12 h. However, our meta-analysis didn’t make definitive conclusions due to the included RCT studies lacking data on 12-h pain scores. Meanwhile, we should notice that the quality evaluation was low and heterogeneity of pain scores after 24 h was high, and the sensitivity analysis we conducted also indicated that the results were not stable, so the final conclusion should be defined by the inclusion of more high-quality RCTS literature in the future. FNB and FICB are the most commonly used regional block techniques for THA, and their effectiveness and safety have been verified [21, 23], but they face the problems of incomplete obturator nerve block and quadriceps muscle movement block, which lead to incomplete postoperative analgesia and the risk of postoperative falls [24–26]. Theoretically, PENG is superior to FNB and FICB because it can block the obturator nerve and is less likely to cause quadriceps block [17, 18]. Our study suggests that it may be as effective as the traditional FICB in sustaining pain relief. Of course, more results are needed to prove this conclusion, and the evaluation of quadriceps strength should also be discussed. It has been reported that patients were unable to perform straight leg elevation after PENG, presenting with quadriceps weakness [27]. Above all, with the continuous improvement of the PENG block technique, it may be an alternative to traditional FICB and femoral nerve blocks in total hip replacement.

### Limitations

Firstly, the five randomized controlled trials had varying numbers of participants, ranging from 60 to 476. Different sample sizes may affect the reliability of statistical results. Secondly, while subgroup and sensitivity analysis have been performed to explore sources of heterogeneity, the high heterogeneity in primary outcome is not well explained. Thirdly, in all five studies, two groups combined with local infiltration analgesia and used different types of anesthesia, although the results of the subgroup analysis did not indicate that there was a source of heterogeneity. Finally, because this is an emerging local analgesia technique, the number of randomized controlled trials is limited, and the data will need to be reevaluated when more high-quality controlled trials are published in the field.

## Conclusion

Pericapsular nerve group block was effective for pain control up to postoperative 6 h and extending the time to the first opioid after THA. Moreover, it reduced postoperative opioid consumption when compared with a placebo group. Due to the high heterogeneity of the pain score after 24 h and the low-quality evidence, more high-quality RCTs are required to draw a definitive conclusion about pain control.

### Supplementary Information

**Additional file 1**. Search strategy in Pubmed.

## Data Availability

Data availability is not applicable to this article as no new data were created or analyzed in this study.

## Abbreviations

PENG: Pericapsular nerve group block
THA: Total hip arthroplasty
RCTs: Randomized controlled trials
PRISMA: Preferred Reporting Items for Systematic Reviews and Meta-Analyses
CI: Confidence interval
WMD: Weighted mean difference
OR: Odds ratio
SA: Spinal anesthesia
GA: General anesthesia
LIA: Local infiltration anesthesia
