# Supplementary material for: The efficacy of pericapsular nerve group block for reducing pain and opioid consumption after total hip arthroplasty: a systematic review and meta-analysis
Source: J Orthop Surg Res. 2024 Apr 8;19:229. doi: 10.1186/s13018-024-04707-x (PMC11000340; doi:10.1186/s13018-024-04707-x)
Supplement: Supplementary file 1 — Additional file 1. Search strategy in Pubmed. [file 13018_2024_4707_MOESM1_ESM.docx]

**Search strategy in Pubmed**

("arthroplasty, replacement, hip"[MeSH Terms] OR ("arthroplasty, replacement, hip"[MeSH Terms] OR ("arthroplasty"[All Fields] AND "replacement"[All Fields] AND "hip"[All Fields]) OR "hip replacement arthroplasty"[All Fields] OR ("arthroplasties"[All Fields] AND "replacement"[All Fields] AND "hip"[All Fields]) OR ("arthroplasty, replacement, hip"[MeSH Terms] OR ("arthroplasty"[All Fields] AND "replacement"[All Fields] AND "hip"[All Fields]) OR "hip replacement arthroplasty"[All Fields] OR ("arthroplasty"[All Fields] AND "hip"[All Fields] AND "replacement"[All Fields]) OR "arthroplasty hip replacement"[All Fields]) OR ("arthroplasty, replacement, hip"[MeSH Terms] OR ("arthroplasty"[All Fields] AND "replacement"[All Fields] AND "hip"[All Fields]) OR "hip replacement arthroplasty"[All Fields] OR ("hip"[All Fields] AND "replacement"[All Fields] AND "arthroplasties"[All Fields]) OR "hip replacement arthroplasties"[All Fields]) OR ("arthroplasty, replacement, hip"[MeSH Terms] OR ("arthroplasty"[All Fields] AND "replacement"[All Fields] AND "hip"[All Fields]) OR "hip replacement arthroplasty"[All Fields] OR ("hip"[All Fields] AND "prosthesis"[All Fields] AND "implantation"[All Fields]) OR "hip prosthesis implantation"[All Fields]) OR ("arthroplasty, replacement, hip"[MeSH Terms] OR ("arthroplasty"[All Fields] AND "replacement"[All Fields] AND "hip"[All Fields]) OR "hip replacement arthroplasty"[All Fields] OR ("hip"[All Fields] AND "prosthesis"[All Fields] AND "implantations"[All Fields]) OR "hip prosthesis implantations"[All Fields]) OR ("arthroplasty, replacement, hip"[MeSH Terms] OR ("arthroplasty"[All Fields] AND "replacement"[All Fields] AND "hip"[All Fields]) OR "hip replacement arthroplasty"[All Fields] OR ("implantation"[All Fields] AND "hip"[All Fields] AND "prosthesis"[All Fields])) OR ("arthroplasty, replacement, hip"[MeSH Terms] OR ("arthroplasty"[All Fields] AND "replacement"[All Fields] AND "hip"[All Fields]) OR "hip replacement arthroplasty"[All Fields] OR ("prosthesis"[All Fields] AND "implantation"[All Fields] AND "hip"[All Fields])) OR ("arthroplasty, replacement, hip"[MeSH Terms] OR ("arthroplasty"[All Fields] AND "replacement"[All Fields] AND "hip"[All Fields]) OR "hip replacement arthroplasty"[All Fields] OR ("replacement"[All Fields] AND "arthroplasties"[All Fields] AND "hip"[All Fields])) OR ("arthroplasty, replacement, hip"[MeSH Terms] OR ("arthroplasty"[All Fields] AND "replacement"[All Fields] AND "hip"[All Fields]) OR "hip replacement arthroplasty"[All Fields] OR ("replacement"[All Fields] AND "arthroplasty"[All Fields] AND "hip"[All Fields]) OR "replacement arthroplasty hip"[All Fields]) OR ("arthroplasty, replacement, hip"[MeSH Terms] OR ("arthroplasty"[All Fields] AND "replacement"[All Fields] AND "hip"[All Fields]) OR "hip replacement arthroplasty"[All Fields] OR ("arthroplasties"[All Fields] AND "hip"[All Fields] AND "replacement"[All Fields]) OR "arthroplasties hip replacement"[All Fields]) OR ("arthroplasty, replacement, hip"[MeSH Terms] OR ("arthroplasty"[All Fields] AND "replacement"[All Fields] AND "hip"[All Fields]) OR "hip replacement arthroplasty"[All Fields] OR ("hip"[All Fields] AND "replacement"[All Fields] AND "arthroplasty"[All Fields])) OR ("arthroplasty, replacement, hip"[MeSH Terms] OR ("arthroplasty"[All Fields] AND "replacement"[All Fields] AND "hip"[All Fields]) OR "hip replacement arthroplasty"[All Fields] OR ("hip"[All Fields] AND "replacement"[All Fields] AND "total"[All Fields]) OR "hip replacement total"[All Fields]) OR ("arthroplasty, replacement, hip"[MeSH Terms] OR ("arthroplasty"[All Fields] AND "replacement"[All Fields] AND "hip"[All Fields]) OR "hip replacement arthroplasty"[All Fields] OR ("replacement"[All Fields] AND "total"[All Fields] AND "hip"[All Fields]) OR "replacement total hip"[All Fields]) OR ("arthroplasty, replacement, hip"[MeSH Terms] OR ("arthroplasty"[All Fields] AND "replacement"[All Fields] AND "hip"[All Fields]) OR "hip replacement arthroplasty"[All Fields] OR ("total"[All Fields] AND "hip"[All Fields] AND "replacements"[All Fields]) OR "total hip replacements"[All Fields]) OR ("arthroplasty, replacement, hip"[MeSH Terms] OR ("arthroplasty"[All Fields] AND "replacement"[All Fields] AND "hip"[All Fields]) OR "hip replacement arthroplasty"[All Fields] OR ("total"[All Fields] AND "hip"[All Fields] AND "replacement"[All Fields]) OR "total hip replacement"[All Fields]) OR ("arthroplasty, replacement, hip"[MeSH Terms] OR ("arthroplasty"[All Fields] AND "replacement"[All Fields] AND "hip"[All Fields]) OR "hip replacement arthroplasty"[All Fields] OR ("total"[All Fields] AND "hip"[All Fields] AND "arthroplasty"[All Fields]) OR "total hip arthroplasty"[All Fields]) OR ("arthroplasty, replacement, hip"[MeSH Terms] OR ("arthroplasty"[All Fields] AND "replacement"[All Fields] AND "hip"[All Fields]) OR "hip replacement arthroplasty"[All Fields] OR ("arthroplasty"[All Fields] AND "total"[All Fields] AND "hip"[All Fields]) OR "arthroplasty total hip"[All Fields]) OR ("arthroplasty, replacement, hip"[MeSH Terms] OR ("arthroplasty"[All Fields] AND "replacement"[All Fields] AND "hip"[All Fields]) OR "hip replacement arthroplasty"[All Fields] OR ("hip"[All Fields] AND "arthroplasty"[All Fields] AND "total"[All Fields]) OR "hip arthroplasty total"[All Fields]) OR ("arthroplasty, replacement, hip"[MeSH Terms] OR ("arthroplasty"[All Fields] AND "replacement"[All Fields] AND "hip"[All Fields]) OR "hip replacement arthroplasty"[All Fields] OR ("total"[All Fields] AND "hip"[All Fields] AND "arthroplasties"[All Fields]) OR "total hip arthroplasties"[All Fields]))) AND ("Nerve Block"[MeSH Terms] OR ("nerve"[All Fields] AND "block"[All Fields]) OR "Nerve Block"[All Fields] OR ("block"[All Fields] AND "nerve"[All Fields]) OR "block nerve"[All Fields] OR ("Nerve Block"[MeSH Terms] OR ("nerve"[All Fields] AND "block"[All Fields]) OR "Nerve Block"[All Fields] OR ("blocks"[All Fields] AND "nerve"[All Fields]) OR "blocks nerve"[All Fields]) OR ("Nerve Block"[MeSH Terms] OR ("nerve"[All Fields] AND "block"[All Fields]) OR "Nerve Block"[All Fields] OR ("nerve"[All Fields] AND "blocks"[All Fields]) OR "nerve blocks"[All Fields]) OR ("Nerve Block"[MeSH Terms] OR ("nerve"[All Fields] AND "block"[All Fields]) OR "Nerve Block"[All Fields] OR ("nerve"[All Fields] AND "blockade"[All Fields]) OR "nerve blockade"[All Fields]) OR ("Nerve Block"[MeSH Terms] OR ("nerve"[All Fields] AND "block"[All Fields]) OR "Nerve Block"[All Fields] OR ("blockade"[All Fields] AND "nerve"[All Fields]) OR "blockade nerve"[All Fields]) OR ("Nerve Block"[MeSH Terms] OR ("nerve"[All Fields] AND "block"[All Fields]) OR "Nerve Block"[All Fields] OR ("blockades"[All Fields] AND "nerve"[All Fields])) OR ("Nerve Block"[MeSH Terms] OR ("nerve"[All Fields] AND "block"[All Fields]) OR "Nerve Block"[All Fields] OR ("nerve"[All Fields] AND "blockades"[All Fields]) OR "nerve blockades"[All Fields]) OR ("Nerve Block"[MeSH Terms] OR ("nerve"[All Fields] AND "block"[All Fields]) OR "Nerve Block"[All Fields] OR ("chemical"[All Fields] AND "neurolysis"[All Fields]) OR "chemical neurolysis"[All Fields]) OR ("Nerve Block"[MeSH Terms] OR ("nerve"[All Fields] AND "block"[All Fields]) OR "Nerve Block"[All Fields] OR ("chemical"[All Fields] AND "neurolyses"[All Fields]) OR "chemical neurolyses"[All Fields]) OR ("Nerve Block"[MeSH Terms] OR ("nerve"[All Fields] AND "block"[All Fields]) OR "Nerve Block"[All Fields] OR ("neurolyses"[All Fields] AND "chemical"[All Fields])) OR ("Nerve Block"[MeSH Terms] OR ("nerve"[All Fields] AND "block"[All Fields]) OR "Nerve Block"[All Fields] OR ("neurolysis"[All Fields] AND "chemical"[All Fields])) OR ("Nerve Block"[MeSH Terms] OR ("nerve"[All Fields] AND "block"[All Fields]) OR "Nerve Block"[All Fields] OR "chemodenervation"[All Fields]) OR ("Nerve Block"[MeSH Terms] OR ("nerve"[All Fields] AND "block"[All Fields]) OR "Nerve Block"[All Fields] OR "chemodenervations"[All Fields]) OR "Nerve Block"[MeSH Terms])
